# Beyond the Prostate: Incidental Detection of Male Breast Carcinoma on [^18^F]DCFPyl

**DOI:** 10.2967/jnumed.125.270683

**Published:** 2026-03

**Authors:** Farzana Z. Ali, Pawan K. Gupta, Martin S. Allen-Auerbach

**Affiliations:** Division of Nuclear Medicine, Department of Molecular and Medical Pharmacology, UCLA, Los Angeles, California

Prostate-specific membrane antigen (PSMA) is consistently expressed in tumor neovasculature but variably in tumor cells, with prior reports demonstrating PSMA uptake in invasive ductal carcinoma in men (1). This case reinforces the value of PSMA in characterizing breast cancer in men, particularly for tumors with low [^18^F]FDG avidity.

A 72-y-old man with prostate cancer (Gleason score, 4 + 3) who underwent radical prostatectomy and chemoradiation presented with an elevated prostate-specific antigen level (149.93 ng/mL). A follow-up PET/CT scan with ^18^F-piflufolastat ([^18^F]DCFPyl) demonstrated intense tracer uptake (SUV_max_, 16.8) at the vesicourethral anastomosis extending along the bladder wall and neck (Fig. 1A), consistent with residual or recurrent disease. [^18^F]DCFPyl uptake (SUV_max_, 7.7) was also noted in his known osseous metastasis in the left superior pubic ramus (Fig. 1B).

**FIGURE 1.**
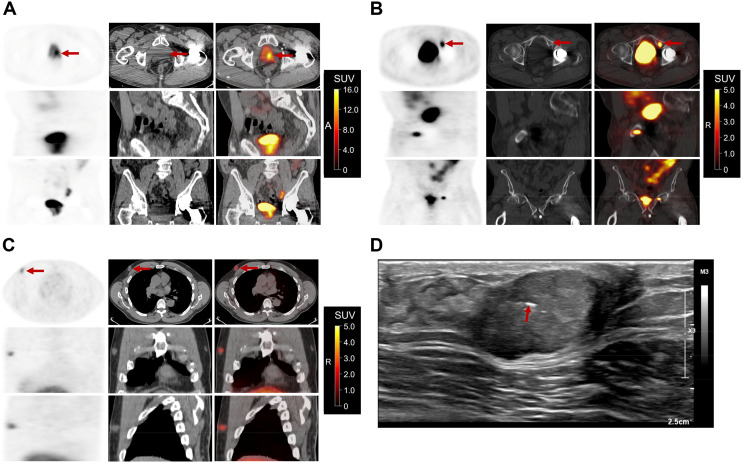
PET, CT, and fused PET/CT images showing [^18^F]DCFPyL uptake in vesicourethral anastomosis extending along bladder wall and neck (A), left superior pubic ramus metastasis (B), and right retroareolar mass (C). (D) Ultrasound-guided biopsy with microclip placement.

Incidentally, moderate [^18^F]DCFPyl uptake (SUV_max_, 2.8) was seen in the retroareolar region of the right breast, corresponding to a subcutaneous ovoid mass on CT, measuring 17 mm (transverse) × 27 mm (anteroposterior) × 17 mm (craniocaudal) (Fig. 1C). Ultrasound-guided core needle biopsy of the mass (Fig. 1D) confirmed a grade 2 invasive ductal carcinoma that was estrogen receptor– and progesterone receptor–positive and human epidermal growth factor receptor 2–negative, with a Ki-67 index of 10%.

This case illustrates atypical PSMA expression in estrogen receptor–positive, low-grade invasive ductal carcinoma. It highlights the potential role of PSMA PET/CT in the evaluation of breast cancer in men, especially those with low [^18^F]FDG-avid tumors. PSMA is expressed in 84% of breast cancer lesions (2) and outperforms [^18^F]FDG PET/CT for detecting distant metastases (3), highlighting the need for broader PSMA imaging in nonprostatic malignancies (4) and validation in larger cohorts.

## DISCLOSURE

No potential conflict of interest relevant to this article was reported.
